# HARNU-Net: Hierarchical Attention Residual Nested U-Net for Change Detection in Remote Sensing Images

**DOI:** 10.3390/s22124626

**Published:** 2022-06-19

**Authors:** Haojin Li, Liejun Wang, Shuli Cheng

**Affiliations:** 1 College of Information Science and Engineering, Xinjiang University, Urumqi 830046, China; lhj96599@stu.xju.edu.cn (H.L.); wljxju@xju.edu.cn (L.W.); 2 College of Mathematics and System Science, Xinjiang University, Urumqi 830046, China

**Keywords:** change detection, remote sensing images, feature fusion, attention mechanism, adjacent strategy, hierarchical structure

## Abstract

Change detection (CD) is a particularly important task in the field of remote sensing image processing. It is of practical importance for people when making decisions about transitional situations on the Earth’s surface. The existing CD methods focus on the design of feature extraction network, ignoring the strategy fusion and attention enhancement of the extracted features, which will lead to the problems of incomplete boundary of changed area and missing detection of small targets in the final output change map. To overcome the above problems, we proposed a hierarchical attention residual nested U-Net (HARNU-Net) for remote sensing image CD. First, the backbone network is composed of a Siamese network and nested U-Net. We remold the convolution block in nested U-Net and proposed ACON-Relu residual convolution block (A-R), which reduces the missed detection rate of the backbone network in small change areas. Second, this paper proposed the adjacent feature fusion module (AFFM). Based on the adjacency fusion strategy, the module effectively integrates the details and semantic information of multi-level features, so as to realize the feature complementarity and spatial mutual enhancement between adjacent features. Finally, the hierarchical attention residual module (HARM) is proposed, which locally filters and enhances the features in a more fine-grained space to output a much better change map. Adequate experiments on three challenging benchmark public datasets, CDD, LEVIR-CD and BCDD, show that our method outperforms several other state-of-the-art methods and performs excellent in F1, IOU and visual image quality.

## 1. Introduction

Change detection (CD) is a remote sensing image interpretation task to obtain a change map by comparing different temporal images of the same geographical region. Monitoring changes in specific areas of the Earth’s surface is crucial for some individuals and institutions to make critical decisions. Therefore, CD has received much attention as a research hotspot in the field of remote sensing. With the quick advancement of remote sensing technology, it has become gradually easier to obtain remote sensing images that can be used for CD, which promoted the development of CD to a certain extent. At present, CD is widely used in land use monitoring [1], disaster assessment [2], urban development planning [3], environmental monitoring [4], and other fields.

Based on the unit of analysis of the model, traditional CD methods can be classified as pixel-based [5] and object-based [6] methods. The pixel-based method usually generates the change map based on the pixel differences between different temporal images. This method requires data to go through several image pre-processing steps, including radiation correction [7], geometric correction [8], etc. For the processed data, the appropriate CD method is used to obtain a set of differential features, and finally generate the change map with a threshold segmentation or clustering method. Celik et al. [5] uses principal component analysis (PCA) [9] extract orthogonal feature vectors, and then implements CD by k-means clustering algorithm [10]. However, the pixel-based method does not fully consider the relationship of feature contextual information during processing, and inevitably leads to the generation of various types of noise and isolated change pixels, which influence the quality of change maps. The object-based method uses structural and geometric information between different temporal images to generate change maps, which largely suppresses the generation of isolated noise, but the method also has high requirements for the registration of different temporal images. These two methods not only require a lot of manual intervention in the image pre-processing stage, but also rely heavily on the experience of professionals for threshold setting. Therefore, there is an immediate requirement to developing automatic and efficient CD algorithms.

In the early years, due to the scarcity of labeled data, research in the field of remote sensing image interpretation started in an unsupervised direction and has continued until now [11,12]. Considering the nature of unsupervised methods, most unsupervised methods use the physical properties of the data. For example, in the field of land cover classification, the texture representation of medium resolution imaging spectrometer data can be used to better reveal the land cover type [13], and in recent studies, Hidden Markov Models have been successfully applied to fully polarized land cover classification [14,15], and good results have been obtained. However, data-driven deep learning methods developed rapidly in various computer vision fields in recent years due to the increasing accessibility of data, such as target detection [16], target tracking [17], super-resolution reconstruction [18],, and semantic segmentation [19], and most of the methods have achieved better results than traditional methods. Therefore CD methods are gradually evolving from traditional methods to deep learning-based methods. Since convolutional neural networks (CNN) [20] are most commonly applied in computer vision tasks, networks based on CNN such as VGG [21], U-Net [22], and ResNet [23] are widely introduced to the field of remote sensing CD to obtain better change maps.

Owing to the resemblance between the CD and the semantic segmentation tasks, early CD models were obtained through simply modification of the semantic segmentation model, so most of them are single-stream structures. Single-stream CD models have been used until now due to their simple modification and smaller computational expenditure. However, the disadvantages of the single-stream CD model were gradually emerged, so the Siamese network [24] was introduced into the CD domain to form the Siamese CD model. Therefore, we divide the deep learning-based remote sensing CD model into single-stream model [25,26,27,28] and Siamese model [29,30,31,32]. In the single-stream CD model, Zheng et al. [25] used U-Net as the backbone network and two three-channel images are concatenated into one six-channel image as input, embedding newly designed cross-layer blocks into the encoder stage of the backbone network, integrating multi-level context information and multi-scale features; Peng et al. [26] concatenated a pair of bi-temporal images and inputs to the U-Net++ network, the global information is used to produce feature maps with higher spatial accuracy, and the multi-level feature maps are then assembled to produce change map with high accuracy. Unlike the single-stream CD model, the bi-temporal images are processed separately by the Siamese backbone, after which the two sets of depth features undergo a series of processing to obtain the change map. Chen et al. [29] proposed a Siamese spatial–temporal attention network, designed a CD self-attentive mechanism to simulate the spatio-temporal relationship between feature context, and finally obtained a better change map. Zhang et al. [30] proposed an image fusion network, highly representative deep features are extracted through a Siamese network, and then inputted the depth features into the differential identification network under deep supervision to obtain the change map.

Although the existing deep learning-based CD approaches outperform most traditional approaches, there are still many limitations in terms of structure and functionality. First, in terms of structure, most models follow a U-shaped codec structure [22]. The problem of back-propagation gradient disappearance is solved to a certain extent using the skip connection mechanism, but because the connection is too sloppy, the features of the two convolution layers being connected have large semantic differences, which leads to increased learning difficulty of the network. Second, in terms of function, the problems of small target miss detection, low robustness to pseudo-change, and irregular edges of the extracted change regions are prevalent on most CD models, and these problems are also urgent problems in CD.

To address the above problem, we propose a model and named it Hierarchical Attention Residual Nested U-Net (HARNU-Net) according to its characteristics. First, the improved U-Net++ [33] is being used as the backbone network for feature extraction, and the four levels of output features extracted by U-Net++ are fed into the Adjacent Feature Fusion Module (AFFM), and the AFFM can combine multi-level features and context information to make the output change map contain more regular change boundaries. Then, the fused four features are fed into the hierarchical attention residual module (HARM) separately. HARM can enhance features in finer-grained space, and effectively suppress problems such as small target miss detection and pseudo-change interference. Finally, the four processed features are concatenated at the channel level and processed to acquire a precise change map.

The major contributions of this paper are as follows:

1. We proposed a novel and powerful network for remote sensing image CD, called HARNU-Net. Compared with the baseline network U-Net++, our network significantly reduces the miss detection rate of small change regions and shows strong robustness on pseudo-change cases.
2. We proposed HARM for effective enhancement of features in a finer-grained space, using the feature transferability of the hierarchy to effectively filter out redundant information and provide powerful feature representation and analysis capabilities for the model. As a plug-and-play module, HARM can be easily transplanted to other models.
3. The AFFM proposed by us can effectively integrate multi-level features and context information, so as to reduce the learning difficulty of the model during the training process, and make the boundary of the output change map more regular.

The rest of the paper is structured as follows. Section 2 introduces the related work of the research. The proposed method is described in detail in Section 3. In Section 4, the comparative experiments between our model and other seven CD models are introduced in detail, and a sequence of ablation experiments verify the reliability and validity of our proposed module. The paper is concluded in Section 5.

## 2. Related Work

The introduction of deep learning into the field of CD accelerated the development of the field. The mature CD model makes people gradually get rid of the tedious task of manually labeling change regions. Since the emergence of Siamese network, because its structural is particularly suitable for CD task, it has been applied into the field of CD by more and more researchers. Zhan et al. [34] first brought the Siamese network to the CD task and proposed a Siamese CD model. At that time, the features extracted by their model were more abstract and robust than those generated by traditional methods. Fully Convolutional Network (FCN) [35], which is widely applied to dense prediction tasks, is also adopted in the CD task. Rodrigo et al. [36] proposed three CD models based on FCN, which are the first end-to-end trainable models proposed in the CD domain. In recent years, variants of FCN such as U-Net and U-Net++ are broadly applied in the field of CD. Peng et al. [26] used U-Net++ to design an end-to-end CD model, which uses multi-level semantic feature map to generate the change map.

Most of the design of CD models focus on the feature extraction phase, while the important feature fusion phase is often ignored or only using coarse feature fusion strategy [26,27,37]. Although a well-designed feature extraction part in a CD model can assist the model obtain more detailed information for CD tasks, the lack of good feature fusion strategy may lead to deterioration of the final CD results. Neural networks as a hierarchical structure network, it is not same in the semantics of output features at different levels. In many works, visualizing the features at different levels of neural networks can help us better understand how the intermediate layers of neural networks work. Typically, low-level features are larger in size and contain more local detail information, but lack the concept of global semantics; high-level features after more convolution layers become smaller in size, thus better summarize the global content of the image. In previous studies, to surmount the lack of detailed and global information in a single feature, researchers have used simple skip connection operations similar to those in FCN and U-Net to integrate high-level features with low-level features. For example, Li et al. [37] proposed a CD model with U-Net structure. Their model only simply connects the features of different semantics. Although the skip connection operation may bring some performance gains to the model, some connections in the simple skip connections not only will not enhance the capabilities of the network, but also may have a negative impact on the network. We propose AFFM to address this issue. According to the adjacency strategy, AFFM performs complementary fusion operations on the features of different layers. This feature fusion approach preserves the feature information at each level, and complements the missing elements of features at various levels from adjacent features. At last it builds the foundation for the final output of the change map with regular boundaries.

When looking at a picture, human beings can quickly find the contents they pay attention to in complex scenes and ignore invalid information. This mechanism for diverting attention to the most important regions of an image and ignoring irrelevant parts is called attention mechanism [38]. Researchers introduced this mechanism to the field of computer vision successfully. In recently, the attention mechanism has shown the great success in various fields, such as image classification [39], semantic segmentation [40], person re-identification [41], etc. In the field of CD, Wang et al. [42] added the fused attention module of channel and spatial features to the decoding part of the proposed CD network, which proved that using the fusion attention module can improve the accuracy of the output results; Chen et al. [43] designed a dual-attentive concatenated FCN network, which showed better capabilities by capturing the long-range dependencies between features, and obtained more discriminative feature representations. Although all of the above methods achieved good results, they all neglected to partially strengthen and weaken the features on a finer-grained space, which led to frequent misses in detecting small change regions. Our HARM employs an attention mechanism based on finer feature segmentation, which effectively enhances the feature representation in fine-grained space and provides excellent performance in small change region detection.

## 3. Methodology

In this section, the general network architecture of HARNU-Net is introduced in Section 3.1, the improved backbone network is introduced in Section 3.2, the proposed adjacent feature fusion module (AFFM) and the hierarchical attention residual module (HARM) are introduced in Section 3.3 and Section 3.4, respectively, and the loss function is introduced in Section 3.5.

### 3.1. Network Architecture

**Figure 1:**
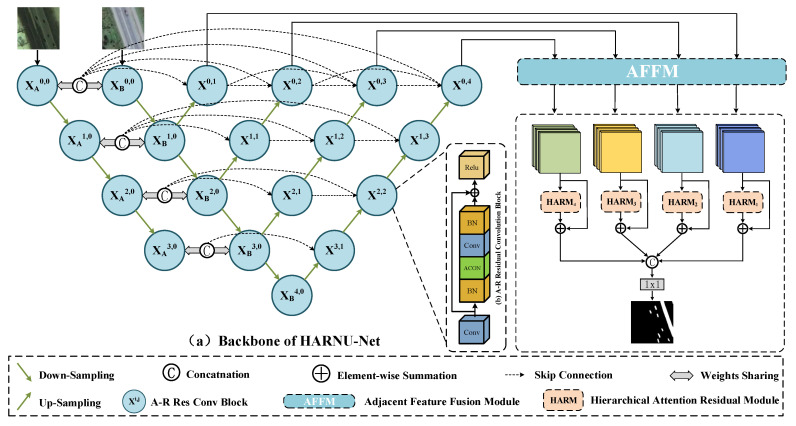
Architecture of the proposed HARNU-Net. (**a**) is the backbone of HARNU-Net, used for feature extraction. (**b**) is an improved convolutional block, proposed to enhance the backbone network performance. AFFM is used for feature fusion, HARM is used for feature filtering and enhancement. Detailed structure of AFFM and HARM are shown in Figure 2 and Figure 3.

The proposed HARNU-Net uses a pair of bi-temporal remote sensing images as input and finally outputs a binary change map. The network uses a standard codec architecture with a series of feature processing modules. The entire network structure can be split into three parts as shown in Figure 1. The general structure of these three parts is as follows:

(1) Feature extraction part: An improved U-Net++ is used as the backbone network, and its encoder part is adjusted to a Siamese structure to meet the bi-temporal images input requirements of the CD task.
(2) Feature fusion part: Unlike the most of previous approach of using only the output features of the last decoder layer of U-Net++, we innovatively use the output features of the four stages of the network to serve the final result. We consider the similarity and complementarity between adjacent features of different layers, so we use AFFM based on the adjacency strategy for complementary fusion of features.
(3) Feature reinforcement part: The four groups of features are reinforced separately using HARM designed by the Convolutional Block Attention Module (CBAM) [44] with a hierarchical structure, and the change maps are output after the final processing.

### 3.2. Improved Backbone Network

The network with codec structure based on U-Net and U-Net++ performs well on various semantic segmentation tasks, and we introduce the U-Net++ into the CD task because of the resemblance between the CD task and the segmentation task. Bi-temporal remote sensing images usually contain many complicated feature information, and the data have high intra-group variability and low inter-group variability [45], which makes it extremely hard to differentiate between changed and unchanged areas in the CD process. Using the dense skip connection mechanism of U-Net++ can better preserve detailed information during feature extraction and avoid confounding effects. Compared with the simple skip connection mechanism of U-Net, the dense skip connection mechanism can avoid the semantic gap caused by long connections that span too large, thus making the learning difficulty of the whole network much less. Given the specificity of the CD task, we embed the Siamese network into the encoder stage of the backbone network. A pair of bi-temporal remote sensing images are sent to two encoder branches to extract image features, and the two branches share the same weight parameter. Using the same convolutional branch for two different images to extract features, and activating weights at the same location in the feature map, which makes the network to effectively distinguish between changed and unchanged areas in subsequent processing.

As shown in Figure 1, the features extracted from two images by convolution blocks of the same layer are concatenated at the channel level, and then concatenated with the features on the decoder through the dense skip connection mechanism. For example, the features $F_{A}^{0,0}$$\in R^{C\times H\times W}$ and $F_{B}^{0,0}$$\in R^{C\times H\times W}$ obtained from a pair of bi-temporal images A,B processed by convolutional blocks $X_{A}^{0,0}$ and $X_{B}^{0,0}$ are first concatenated at the channel level to form the bi-temporal feature $F^{0,0}$$\in R^{2C\times H\times W}$, and then $F^{0,0}$ is concatenated with the feature $F_{B}^{1,0}$$\in R^{C\times H\times W}$ output from convolutional block $X_{B}^{1,0}$ at the channel level and fed to convolutional block $X^{0,1}$ to generate the first stage feature $F^{0,1}$$\in R^{C\times H\times W}$. Since the pooling operation is included in each stage of the encoding operation to condense the information of the features, $F_{B}^{1,0}$ is upsampled to comply with the concatnation specification before the channel fusion operation with $F^{0,0}$.

It is also worth saying that assuming that each node $X^{i,j}$ of U-Net++ is called a convolutional block, we modified the internal structure of the convolutional block. As shown in Figure 1b, we designed the convolutional block as a residual structure [23] and introduced the ACON activation function [46] in it, the modified convolutional block can adaptively choose whether to activate specific neural nodes or not, effectively improving the overall performance of the network. We call this convolutional block ACON-Relu Residual Convolutional Block (A-R). Let $F^{i,j}$$\in R^{C\times H\times W}$ be the output of A-R $X^{i,j}$, where *i* indicates the number layers of down-sampling layers and *j* indicates the number layers of up-sampling layers, then the formula for $F^{i,j}$ is as follows:

(1) Res(·)=R(B(C(A(B(C(·)))))+C(·))

(2) $$F^{i,j}=\left\{\begin{array}{cc} M\left(\mathrm{Res}\left(x^{i-1,j}\right)\right) & j=0\\ \mathrm{Res}\left(\left[x_{A}^{i,0},x_{B}^{i,0},DC\left(x^{i+1,j-1}\right)\right]\right) & j=1\\ \mathrm{Res}\left(\left[x_{A}^{i,0},x_{B}^{i,0},{\left[x^{i,k}\right]}_{k=1}^{j-1},DC\left(x^{i+1,j-1}\right)\right]\right) & j>1 \end{array}\right.$$

where the function Res(·) represents the overall operation of the A-R, *R*(·) represents the Relu activation operation, *B*(·) represents the Batch Normalization operation, *C*(·) denotes the convolutional operation, *A*(·) denotes the ACON activation operation, *M*(·) denotes the max pooling down-sampling operation, *DC*(·) denotes the deconvolution up-sampling operation, and [ , ] denotes the channel concatenation operation for the features in it. When *j* = 0, it means that the input features are downsampled in the encoder stage to extract features with higher-dimensional information, and when *j* > 0, it means that the features between adjacent levels are fused to generate more complete features containing local detail information and global semantic information.

In our proposed backbone network, the A-R in the encoder stage further deepens the features by gradually expanding the number of channels of the features, and the A-R in the decoder stage further condenses the features by gradually reducing the number of channels of the features. The size of the features output from the A-R located at the same hierarchical position is the same. The down-sampling operation halves the height and width of the feature map and keeps the number of channels constant, and the up-sampling operation doubles the height and width of the feature map and keeps the number of channels constant. Table 1 lists the feature map size information for each A-R input and output of the whole backbone network.

### 3.3. Adjacent Feature Fusion Module

The advancement of remote sensing CD in recent years is to some extent due to the research of researchers in various feature fusion strategies. However, many existing feature fusion methods underestimate the significance of semantic association [47], ignore the semantic differences between features when performing the integration of high-level and low-level features, which can lead to irrelevant noise in the fused features and thus impact the precision of the change map. To better solve computer vision tasks, we need to make our final extracted features contain both local detail information and global semantic information, and it is difficult to satisfy this requirement using only single-level features output from deep networks. Considering this situation, we propose AFFM, which innovatively uses the four level features derived from the backbone network for feature fusion, and then passes them to the next module for further processing. AFFM focuses on the complementary fusion between adjacent features, which achieves feature complementarity and mutual spatial enhancement between adjacent features, greatly reduces the overall learning difficulty of the network.

**Figure 2:**
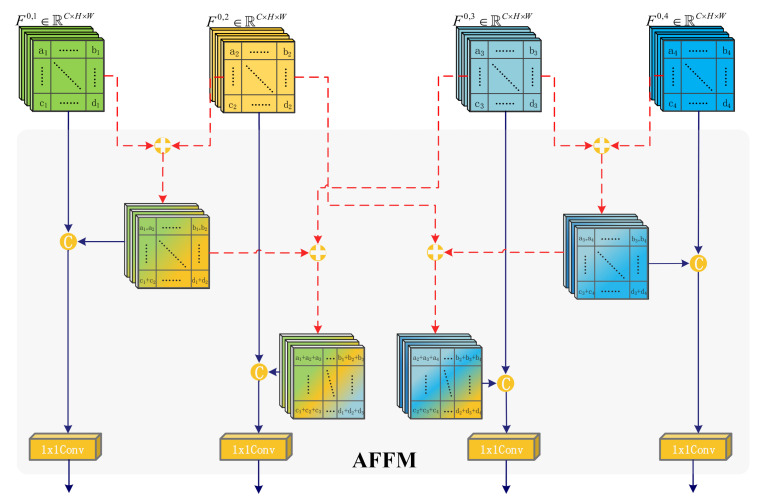
Architecture of Adjacent Feature Fusion Module (AFFM).

For *N* input feature maps *F* = {$f_{i},i=1,2,...,N$}, AFFM will generate N fused feature maps of the same shape $F^{r}$ = {$f_{i}^{r},i=1,2,...,N$}. As shown in Figure 2, AFFM first sums the elements of adjacent features, after which the obtained preliminary fused features are concatenated at the channel level with the original features, and finally the combined features are output after feeding into the 1 × 1 convolutional transform channel. Since the final output of the CD task is a binary change map, we think that the element-wise summation operation in AFFM can effectively alleviate the coarseness of each layer of features using the correlation between different layers of features, and the channel re-fusion with the original features prevents the loss of effective features, and finally achieves the effective fusion of details and semantic information of different layers of features. The specific operation formula of AFFM is shown as follows:

(3) $$f_{i}^{r}=\left\{\begin{array}{cc} C\left(\left[f_{i}⊕f_{i+1},f_{i}\right]\right) & i=1\\ C\left(\left[f_{i-1}⊕f_{i}⊕f_{i+1},f_{i}\right]\right) & 1<i<N\\ C\left(\left[f_{i-1}⊕f_{i},f_{i}\right]\right) & i=N \end{array}\right.$$

where C(·) denotes 1 × 1 convolution, ⊕ denotes element-wise summation, and [] denotes a channel concatenation operation for the features in it. In short, AFFM first makes the initial fusion of two or three adjacent features by element-wise summation, so that the features focus more on the common part elements and complement the missing part elements, then performs channel fusion with the original features to prevent the loss of useful features, and finally adjusts the channels after 1×1 convolution before outputting.

### 3.4. Hierarchical Attention Residual Module

**Figure 3:**
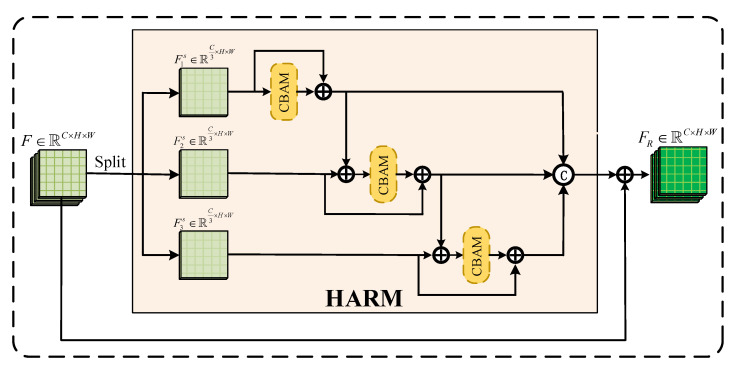
Architecture of Hierarchical Attention Residual Module (HARM).

The HARM is the key innovation and main component of HARNU-Net. It is responsible for finer filtering and reinforcement of the features output from AFFM. Most attentional CD networks in the past have been obsessed with filtering and reinforcing features at the feature level [30,42], inspired by Res2Net [48], as shown in Figure 3, we propose HARM by reinforcing features at a finer scale. In the process of network transmission, features contain a lot of low-dimensional and high-dimensional information, but not all the information contained in features is conducive to the CD [47,49], and if the invalid information in the features is not removed well in the CD process, it will exponentially grow the difficulty of training the network [44]. Therefore, we introduce CBAM in the design of HARM, which consists of a channel attention module (CAM) and a spatial attention module (SAM) together.

**Figure 4:**
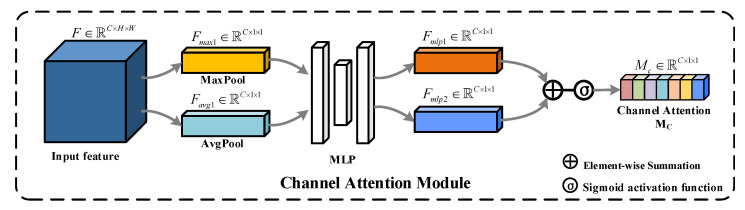
Architecture of CAM.

Channel attention module: The core idea of this module is to produce a channel attention map using the relationship between feature channels. We can use the channel attention map to recalibrate the weights of the corresponding features at the channel level. As shown in Figure 4, CAM first performs adaptive max pooling and adaptive average pooling with output scale of 1, respectively, on the input features *F*$\in R^{C\times H\times W}$ to aggregate spatial information, and this operation will yield two C-dimensional pooling features $F_{max1}$$\in R^{C\times 1\times 1}$ and $F_{avg1}$$\in R^{C\times 1\times 1}$, which are then fed to a shared multilayer perceptron (MLP), and the two features are output after element summation and Sigmoid activation to generate the required channel attention map $M_{c}$$\in R^{C\times 1\times 1}$. In short, the CAM is computed as follows:

(4) $$M_{c}\left(F\right)=\sigma \left(\mathrm{MLP}\left(\mathrm{AvgPool}\left(F\right)\right)⊕\mathrm{MLP}\left(\mathrm{MaxPool}\left(F\right)\right)\right)$$

where F denotes the input features, ⊕ denotes the element-wise addition, σ denotes the Sigmoid activation function, and the MLP weights are shared. Using the channel attention map, the features can be refined at the channel level by multiplying the channel attention map with the corresponding features, the effect of suppressing irrelevant channels and strengthening relevant channels can be achieved, and the globally optimized features can be obtained at the channel level. 

**Figure 5:**
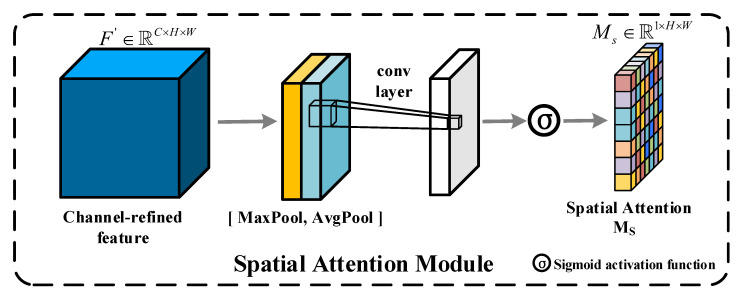
Architecture of SAM.

**Spatial attention module**: The core idea of this module is to produce a spatial attention map using the spatial association of the feature. Unlike channel attention, applying spatial attention allows recalibrating the weights at the spatial level of the corresponding features. As shown in Figure 5, SAM first performs maximum pooling and average pooling to the input features $F^{\prime }$$\in R^{C\times H\times W}$ along the channel direction, and this operation will yield two two-dimensional features $F_{max2}$$\in R^{1\times H\times W}$ and $F_{avg2}$$\in R^{1\times H\times W}$, after which these two feature maps are fed into a layer of convolution after concatenation at the channel level, and finally activated by Sigmoid to obtain the desired spatial attention map $M_{s}$$\in R^{1\times H\times W}$. In short, the SAM is computed as follows:

(5) $$M_{s}\left(F^{\prime }\right)=\sigma \left(f^{7\times 7}\left(\left[\mathrm{AvgPool}\left(F^{\prime }\right);\mathrm{MaxPool}\left(F^{\prime }\right)\right]\right)\right)$$

where $F^{\prime }$ denotes the input features, [ ; ] denotes the channel concatenation, σ denotes the Sigmoid activation function, and $f^{7\times 7}$ denotes the convolution operation with a convolution kernel size of 7 × 7. The weight value of each pixel position can be adjusted by multiplying the 2D features generated by the spatial attention module with the corresponding features, and the spatial layer of the final output feature map will be adaptively tailored to fit the task. In short, in the CD task, the position weights of the unchanged pixels of the SAM-processed features will be weakened and the position weights of the changed pixels will be strengthened, so that the network can be better suited to the needs of the CD task.

**Figure 6:**
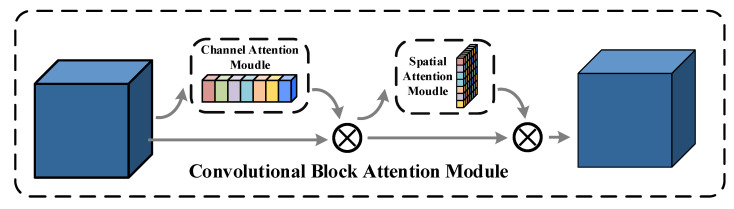
Architecture of CBAM.

As shown in Figure 6, CBAM consists of CAM and SAM in order, not only tells the network which channel location information and spatial location information to focus on the features, but also suppresses or ignores irrelevant channel location information and spatial location information, enhances the feature representation of the model at key locations, and reduces the negative impact of redundant information on the model in making decisions. This attention-based feature enhancement approach allows the network to autonomously explore the optimal representation of the input features at the channel and spatial levels, and the CBAM is calculated as follows:

(6) $$CBAM\left(F\right)=M_{s}\left(M_{c}\left(F\right)⊗F\right)⊗\left(M_{c}\left(F\right)⊗F\right)$$

where *F* denotes the input features and ⊗ denotes the element-wise multiplication. $M_{c}\left(F\right)$ and $M_{s}\left(F\right)$ denote the corresponding channel attention map and spatial attention map generated based on the features *F*, respectively.

In HARM, we divide the input feature mapping *F*$\in R^{C\times H\times W}$ with *C* channels equally into *X* feature mapping subsets at the channel level, denoted by $F_{i}^{s}$$\in R^{\frac{C}{X}\times H\times W}$, where *i*∈{1,2,...,X}. Each $F_{i}^{s}$ has the same space size as *F* except that the channels become C/X. We apply the same CBAM processing to each $F_{i}^{s}$, and with a residual structure to guarantee that the useful information in the original features is not easily and blindly removed. The output produced by $F_{i}^{s}$ after processing is denoted by $Y_{i}^{s}$$\in R^{C/X\times H\times W}$, where *i*∈{1,2,...,X}. Except for $F_{1}^{s}$, all other $F_{i}^{s}$ receive a subset of the feature mapping processed in the previous layer as an extension of the information in that subset of the layer before being processed by CBAM. After the enhancement process of all feature mapping subsets is completed, we will concatenate *X* output features and the final output obtained is represented by $F_{R}$$\in R^{C\times H\times W}$. The specific formula of HARM is as follows:

(7) $$y_{i}^{s}=\left\{\begin{array}{cc} \mathrm{CBAM}\left(F_{i}^{s}\right)⊕F_{i}^{s} & i=1\\ CBAM\left(F_{i}^{s}⊕\left(\mathrm{CBAM}\left(F_{i-1}^{s}\right)⊕F_{i-1}^{s}\right)\right)⊕F_{i}^{s} & i>1 \end{array}\right.$$

(8) $$F_{R}=\left[y_{1}^{s},y_{2}^{s},\ldots ,y_{i}^{s}\right]\;i\in \left\{1,2,\ldots ,X\right\}$$

where CBAM (·) denotes the sequential channel and spatial enhancement of features, ⊕ denotes element-wise summation, and [] denotes the concatenation of feature mapping subsets at the channel level. By the above operation, the output feature $F_{R}$ of HARM contains the content of the change map after being finely processed. Through extensive experiments, it is found that HARM performs best when the number of branches is 3, i.e., *X* = 3.

### 3.5. Loss Funtion

Remote sensing images CD is a typical binary classification task, and applying cross-entropy loss is a common practice for most binary classification tasks. However, in the CD task, there is a serious disequilibrium among the number of changed pixels and the unchanged pixels [50], and often the number of unchanged pixels is much greater than the number of changed pixels, which leads to the fact that CD networks applying general cross-entropy loss will seriously favor a certain class of pixels during the training process [51], making it harder to update the network to the global optimum. Therefore, more consideration is now given to applying weighted cross-entropy loss, which can better balance the class imbalance problem in CD tasks by applying different weight values to the classes. In addition, the dice coefficient loss, which is more applied in the medical image segmentation field [52,53], can also weaken the impact caused by the category imbalance problem better. Based on the above considerations, we choose to use the hybrid loss function of Fang et al. [54] to optimize our network parameters in the training process. This hybrid loss function is composed of both weighted cross-entropy loss and dice coefficient loss. Formally, this loss function is determined by the equation:

(9) $$L=L_{wce}+L_{\mathrm{dice}\;}$$

To describe the weighted cross-entropy loss in more detail, we consider the pixel points in the predicted change map as a set $\hat{Y}$, which can be expressed as [54]:

(10) $$\hat{Y}=\left\{{\hat{y}}_{1},{\hat{y}}_{2},\ldots ,{\hat{y}}_{k}|k=1,2,\ldots ,H\times W\right\}$$

where ${\hat{y}}_{1}$ denotes a pixel point in $\hat{Y}$, *H* and *W* denote the height and width of $\hat{Y}$, consistent with the size of the bi-temporal image pair of the input network. The weighted cross-entropy is expressed in detail as [54]:

(11) $$L_{wce}=\frac{1}{H\times W}\sum_{k=1}^{H\times W}\;\mathrm{weight}\;\left[\;\mathrm{class}\;\right]\cdot \left(\log\left(\frac{\exp(\hat{y}\left[k\right]\left[\;\mathrm{class}\;\right])}{\sum_{l=0}^{1}\exp\left(\hat{y}\left[k\right]\left[l\right]\right)}\right)\right)$$

the value of “class” is 0 or 1, which corresponds to unchanged and changed pixels, respectively. In addition, the definition of dice coefficient loss can be expressed as follows [54]:

(12) $$L_{\mathrm{dice}\;}=1-\frac{2\cdot Y\cdot \mathrm{softmax}(\hat{Y})}{Y+\mathrm{softmax}(\hat{Y})}$$

where *Y* denotes the ground truth.

## 4. Experiment and Analysis

### 4.1. Datasets and Pre-Processing

Training deep CD models requires a lot of labeled images, and large and challenging CD datasets are indispensable for the sake of accurately proving the effectiveness of the model. To evaluate the reliability and validity of our model, we performed a sequence of experiments on three well-known and frequently used CD datasets. These three datasets and the pre-processing are described in detail below.

**Figure 7:**
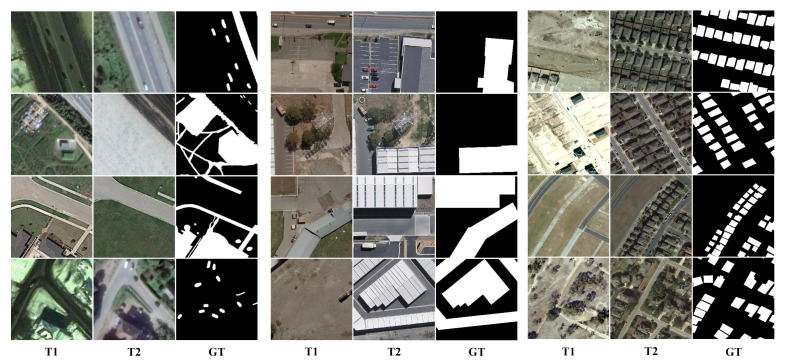
Illustration of samples from three dataset. The samples from left to right are in order from CDD, BCDD, LEVIR-CD. T1 and T2 indicate the bi-temporal image pairs. GT indicates the ground truth.

(1) Change Detection Dataset (CDD) [55]: The dataset contains 11 bi-temporal image pairs obtained from Google Earth (DigitalGlobe) with seasonal changes, of which seven image pairs have a size of 4725 × 2700 pixels and four image pairs have a size of 1900 × 1000 pixels with a resolution of 0.03-1 m/pixel. As shown in Figure 7, the change objects include various size objects, such as buildings, roads, cars, etc. We used 16,000 image pairs of size 256 × 256 generated from these 11 bi-temporal image pairs by cropping and rotating as data for the experiments, of which 10,000 pairs were devoted to training, 3000 pairs to validation, and 3000 pairs to testing.

(2) Building Change Detection Dataset (BCDD) [56]: The dataset contains 1 bi-temporal image pair of size 32,507 × 15,354 pixels acquired by QuickBird satellite, with the acquisition location in Christchurch, New Zealand. In the study area included in this aerial image pair, a magnitude 6.3 earthquake occurred in February 2011, so there was a large growth of large sparse buildings in the following years, bi-temporal image pairs were collected before and after the earthquakes. The authors downsampled the resolution from 0.075 m/pixel to 0.3 m/pixel to facilitate the study. As shown in Figure 7, We cropped this image pair without overlap into 7434 image pairs of size 256 × 256 and randomly assigned these image pairs to the training, validation, and test sets in a ratio of 8:1:1.

(3) LEarning, VIsion and Remote sensing-Change Detection (LEVIR-CD) [29]: The dataset contains 637 pairs of images collected by the authors through Google Earth API in 20 different areas of several cities in Texas, USA, which have a size of 1024 × 1024 pixels and a resolution of 0.5 m/pixel. As shown in Figure 7, LEVIR-CD focuses on small and dense building variations and includes many pseudo-changes caused by season and light, which helps to validate the reliability of the model. For the experiments, considering the limitation of computer GPU memory, we cropped the original image pairs into 10,192 non-overlapping image pairs of size 256 × 256, and used 7120 pairs were devoted to training, 2048 pairs to validation, and 1024 pairs to testing according to the random allocation principle.

Detailed information on the above three CD datasets can be found in Table 2. We also collected the number of changed and unchanged pixels contained in the three datasets and calculated the ratio, and it can be seen that the ratio between the two types is very disparate, the phenomenon is common in the CD datasets.

### 4.2. Evaluation Metrics and Implementation Details

To estimate the capability of our proposed HARNU-Net, we use five evaluation metrics, Precision (Pre), Recall, F1-Score (F1), Intersection over Union (IoU) and Overall Accuracy (OA), all in the range of [0, 1], with larger values denoting higher performance.. The expressions of these five metrics are as follows:

(13) $$\;\mathrm{Pre}\;=\frac{TP}{TP+FP}$$

(14) $$\;\mathrm{Recall}\;=\frac{TP}{TP+FN}$$

(15) $$F1=\frac{2\mathrm{Pre}\cdot Recall}{\mathrm{Pre}+Recall}$$

(16) $$\;\mathrm{IoU}\;=\frac{TP}{TP+FP+FN}$$

(17) $$\;\mathrm{OA}\;=\frac{TP+TN}{TP+TN+FP+FN}$$

where TP, FP, TN, FN denote true positive, false positive, true negative and false negative, respectively. In the CD task, the fewer false detections in the predicted results, the higher the Pre; the fewer missed results, the higher the Recall, but it is difficult to achieve both high Pre and high Recall, and OA indicates the overall classification correctness. Both F1 and IoU are comprehensive metrics for evaluating the CD model, where F1 balances the conflict by considering both Pre and Recall [56], and IoU is the ratio of the intersection of the predicted result and ground truth to the concatenated set [57]. Higher values of F1 and IoU indicate better overall performance of the model.

Our proposed model is realized under the pytorch framework. All experiments we performed were made on an Intel Xeon Gold 5120 @ 2.20 GHz and an NVIDIA TITAN RTX (24 GB). Considering the limitation of GPU memory, in the process of model training, batch size is fixed to 12, Adam [58] is applied as the optimizer, the initial learning rate is set to 0.001 and decreases by half after every 10 epochs. In order to prevent over fitting, epoch is set to 100. The weight values of the network are initialized by Kaiming normalization [59].

### 4.3. Analysis of Experimental Results

#### 4.3.1. Comparison Methods

To verify the superior performance of our proposed methods, seven state-of-the-art CD methods were chosen for comparison on three datasets, and a brief description of the selected methods is given below:

(1) FC-EF [36]: The bi-temporal images are sent to the network as one image after early fusion, and the overall structure follows the idea of FCN with a four skip connection structure. The first layer of the network has 16 output channels, and the number of channels is doubled layer by layer in the encoder stage and then halved layer by layer in the decoder stage.

(2) FC-Siam-conc [36]: The encoder of the FCN network is transformed into a Siamese structure, and the two branches of the encoder stage share weights. The features of the two encoder stages are connected to the features of the decoder through four skip connection to fully fuse the features at different levels.The rest of the structure is the same as FC-EF.

(3) FC-Siam-diff [36]: The structure is the same as that of FC-Siam-conc, with the difference that the features connected to the decoder features are the differential features obtained by the difference of the two encoder features. Therefore, this network has one third less channels than FC-Siam-conc for the decoder at the same stage.

(4) CDNet [60]: A single-stream FCN network architecture consisting of four systolic blocks and four extension blocks with Soft-max classifier. The network is structured as a conventional codec structure with skip connection to efficiently combine coarse and fine information. Notably, the network used a convolution operation with a kernel size of 7 to extract features, and always kept the channels at 64 in the middle layer of the network.

(5) STANet [29]: Two Resnet18-based branching networks are used for extracting the features of bi-temporal image pairs, then the two features are fed into the spatial-temporal attention module to generate spatial-temporal relationship features used to describe contextual features, and finally the output features are fed into the metric module to generate the final change map.

(6) BIT [61]: Proposed a bi-temporal images transformer(BIT) to effectively model the spatial-temporal domain within the context, and pioneered the introduction of transformer into the field of CD. The network replaces the last convolution stage of ResNet18 with BIT, after which refined features are fed to the prediction head to generate change maps.

(7) SNUNet [54]: Uses a densely connected Siamese nested U-shape network to effectively reduce the loss of deep localization information, and designs an integrated channel attention module to further optimize the processing of features.

The main difference between our method and the above seven methods is that we fully use the output features from the four stages of Nested U-Net, while the other methods only use the features output from the last decoder, and on top of that we apply HARM to the four features for a fine-grained feature-enhanced representation, all these operations facilitate us to output a better change map.

Table 3 summarizes the main features of these seven models. For the sake of experimental fairness, we use a set of public codes to implement the above CD models, and all experiments are conducted in the same experimental environment with loss functions and batch sizes set as described in the original literature. It is notable that we choose SNUNet/48, the best performing SNUNet network, as the comparison method.

#### 4.3.2. Analysis Experiments on CDD

Quantitative Analysis: To assess the superiority of our proposed method, we did a comparison with seven other CD methods on the CDD dataset, and the main metrics F1 and IoU showed an improvement of 0.43% to 41.73% and 0.82% to 56.18%, respectively, and Table 4 presents the results of the quantitative comparison. Clearly, our HARNU-Net achieves the best results in all metrics, shows the superior performance of our model. In specific analysis, the earlier FC-EF, FC-Siam-conc and FC-Siam-diff are no longer adaptable to scenarios with more complex changing objects due to their simpler structures and extracting features only by convolution operation. From the results, it can be seen that these three methods are seriously unbalanced in terms of Pre and Recall, resulting in poor results. Similarly, CDNet also suffers from this problem and thus leads to low metrics. STANet greatly reduces the missed detection rate of changed objects with its spatio-temporal attention module, but it also incorrectly identifies many unchanged regions as changed regions resulting in low Pre and thus affecting the main metrics. Both BIT and SNUNet have good metrics, but their IoU metrics are low due to problems such as too coarse handling of features. Our HARNU-Net actively avoids the above errors and thus performs well in dealing with scenarios with multiple classes of changed objects.

**Figure 8:**
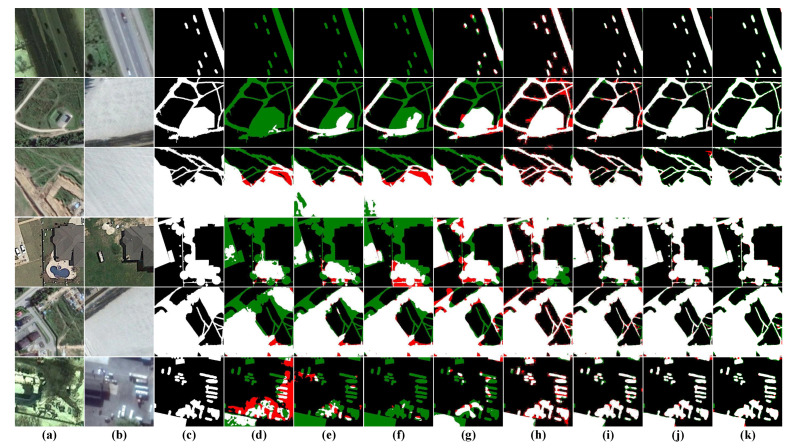
Visualization results on the CDD dataset. (**a**) T1 images. (**b**) T2 images. (**c**) Ground Truth. (**d**) FC-EF. (**e**) FC-Siam-conc. (**f**) FC-Siam-diff. (**g**) CDNet. (**h**) STANet. (**i**) BIT. (**j**) SNUNet. (**k**) Ours. White indicates correctly detected changed areas, black indicates correctly detected unchanged areas, red indicates incorrectly detected unchanged areas as changed areas, and green indicates unpredicted changed areas.

Visual Analysis: To contrast the results more visually, we list the visualization results of eight CD methods, including our method, in Figure 8. To more fully assess the capabilities of each model, we chose six different scenarios to test the models, specifically sparse cars (first row), irregular fine roads (second row), large area changes with fine roads (third row), irregular buildings (fourth row), large buildings with fine roads (fifth row), and dense cars (sixth row). The visualization results show that our results give the best visual results, especially excellent on fine targets and irregular targets, which can better avoid the miss detection of small changed areas and present the boundaries of irregular targets completely.

#### 4.3.3. Analysis Experiments on BCDD

Quantitative Analysis: The results of the presentation of our method on the BCDD dataset compared with the other seven methods in Table 4. It is clear that our method yields the best results in all metrics, with improvements of 2.03% to 40.27% and 3.32% to 49.24% in the main metrics F1 and IoU, respectively, which are mainly attributed to the finer-grained enhancement of the features by HARM. From the results we can see that the FCN-based FC-EF, FC-Siam-conc and FC-Siam-diff perform poorly, indicating that the network without the deeply improved FCN structure is no longer well adapted to today’s CD tasks. The CDNet achieves the second best result in terms of Pre, but also misses many pixels resulting in a low Recall. STANet’s F1 achieves the second best result, which may be attributed to the fact that its PAM focuses more on multi-scale information. SNUNet has a good balance among the metrics, but its lack of focus on feature enhancement in the spatial domain. Although our model yields the best results on all metrics, the IoU is only 82.93%, which indicates that the BCDD dataset is still challenging.

**Figure 9:**
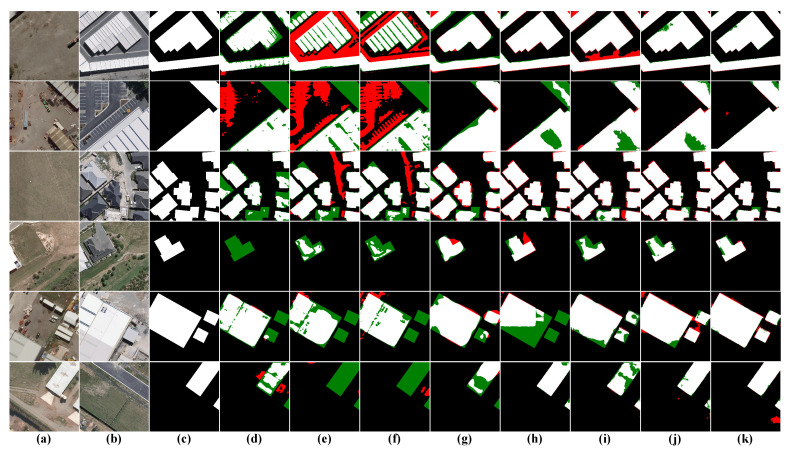
Visualization results on the BCDD dataset. (**a**) T1 images. (**b**) T2 images. (**c**) Ground Truth. (**d**) FC-EF. (**e**) FC-Siam-conc. (**f**) FC-Siam-diff. (**g**) CDNet. (**h**) STANet. (**i**) BIT. (**j**) SNUNet. (**k**) Ours. White indicates correctly detected changed areas, black indicates correctly detected unchanged areas, red indicates incorrectly detected unchanged areas as changed areas, and green indicates unpredicted changed areas.

Visual Analysis: The BCDD dataset mainly concerns the variation in large sparse buildings, but several different types of changed buildings were also chosen to fully assess the capabilities of the model under various conditions, as shown in Figure 9 for visual analysis. The dense regular building (third row) and the small independent building (fourth row) were selected specifically to test the model’s ability to handle regular boundaries and detect small independent targets. From the results of the first row, it can be seen that the three FCN-based structures FC-EF, FC-Siam-conc and FC-Siam-diff fail to adapt to the changed scenarios of large buildings, showing problems such as pretzel noise and incorrect detection of roof texture; CDNet can roughly detect the changes of buildings, but the boundary of its prediction map is very uneven; STANet and SNUNet also have different degrees of omission and misdetection. In particular, the visualization result in the fourth row, the roof of the changed building is almost indistinguishable from the neighboring roads due to the effect of lighting when shooting, so it is a great challenge for the performance of the model. All models except ours fail to detect the changing contours of small independent buildings well, which further illustrates the superiority of HARNU-Net performance.

#### 4.3.4. Analysis Experiments on LEVIR-CD

Quantitative Analysis: We compared our method with seven other methods on the LEVIR-CD dataset. These methods include STANet, and the LEVIR-CD dataset was proposed by the very authors of STANet in [29]. As observed from Table 4, although our model does not achieve optimal results on Pre and Recall, the main metrics F1 and IoU outperform the other methods and have an improvement of 0.06% to 16.88% and 0.11% to 24.3%, respectively. STANet achieves the best results on the Recall, but also has more false detections as a result, as shown by the low Pre. BIT achieves the best result in Pre and the second best result in F1, which should be attributed to the superiority of its transformer module in handling dense small targets, and BIT’s metrics are extremely close to ours. HARNU-Net takes full consideration of the fusion of multi-scale features, and with the fine enhancement of features by HARM, it shows a superior performance in CD of dense small targets.

**Figure 10:**
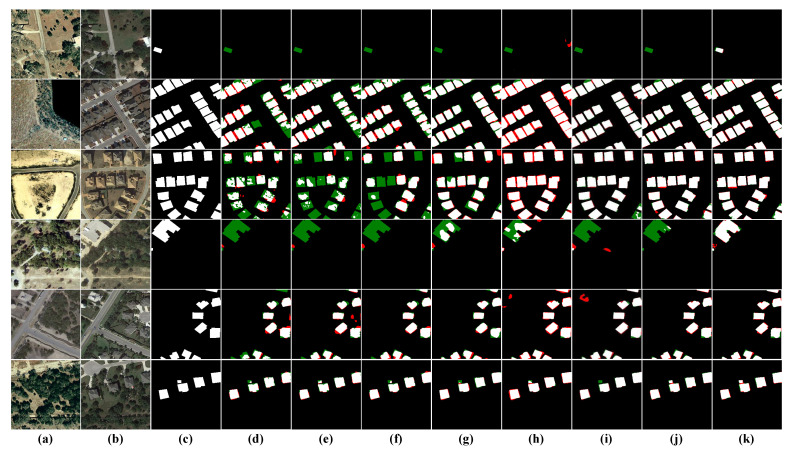
Visualization results on the LEVIR-CD dataset. (**a**) T1 images. (**b**) T2 images. (**c**) Ground Truth. (**d**) FC-EF. (**e**) FC-Siam-conc. (**f**) FC-Siam-diff. (**g**) CDNet. (**h**) STANet. (**i**) BIT. (**j**) SNUNet. (**k**) Ours. White indicates correctly detected changed areas, black indicates correctly detected unchanged areas, red indicates incorrectly detected unchanged areas as changed areas, and green indicates unpredicted changed areas.

Visual Analysis: To show the superiority of our model for the small target detection and dense target boundary delineation, we visualized and analyzed the results of these eight models, as shown in Figure 10. Again, we select some independent small target (first and fourth rows) and sparse small target (fifth and sixth rows) samples considering the comprehensiveness of the test samples. Obviously, the results in the first and fourth rows show that all methods except ours are unsuccessful in detecting changes in independent small targets, and our method also delineates regular change boundaries for small targets. The visualization result in the second and third rows demonstrate that although most methods can detect changes in dense buildings, they all have some problems in boundary delineation. In contrast to other methods, HARNU-Net has more clear and more precise boundaries. Although BIT and SNUNet perform well in some scenes, they still have misdetection in the face of pseudo-change caused by illumination, so their performance in terms of robustness still falls short of our method.

### 4.4. Ablation Study

**Figure 11:**
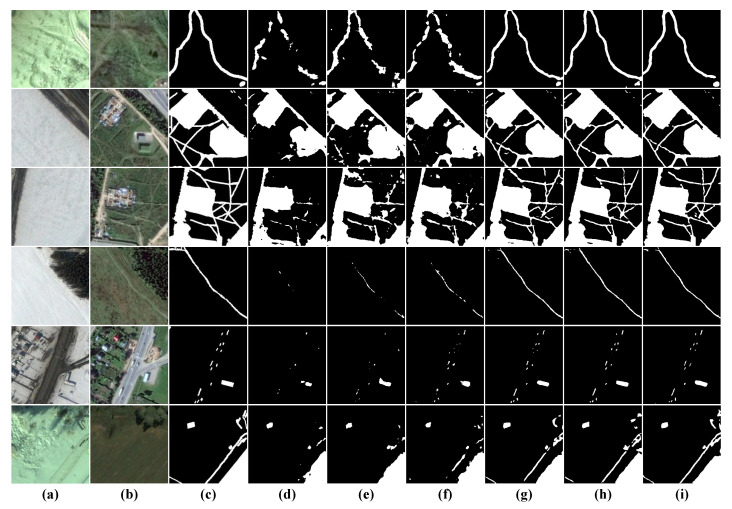
Visualization results of ablation experiments performed on CDD dataset. (**a**) T1 images. (**b**) T2 images. (**c**) Ground Truth. (**d**) Baseline. (**e**) Baseline + A-R. (**f**) Baseline + AFFM. (**g**) Baseline + HARM. (**h**) Baseline + AFFM + HARM. (**i**) Baseline + A-R + AFFM + HARM. White indicates the predicted change area, black indicates the predicted unchanged area.

**Figure 12:**
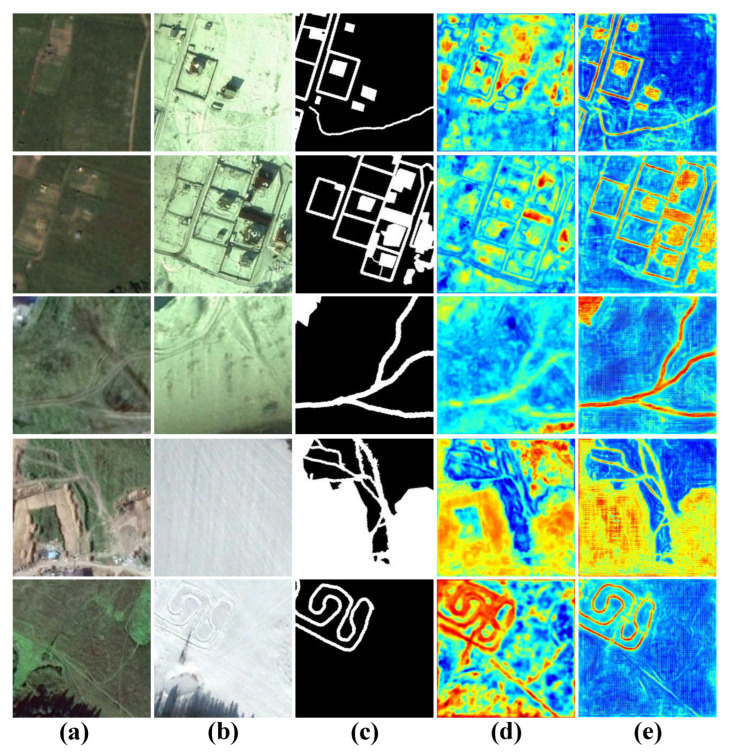
Comparison of attentional heat maps before and after baseline model improvement. (**a**) T1 image, (**b**) T2 image, (**c**) ground truth, (**d**) attentional heat map generated by the baseline model UNet++, (**e**) attentional heat map generated by HARNU-Net.

To assess the usefulness of each module proposed in our paper, we conducted a sequence of ablation experiments on the CDD dataset. Specifically, we use the unmodified U-Net++ network as the baseline and only the output of $X^{0,4}$ as the source of the final results; the other ablation experiments are baseline+A-R, baseline+AFFM, baseline+HARM, baseline+AFFM+HARM, and baseline+A-R+AFFM+HARM. To show the role of each module more visually, we visualize the results of the six experiments in Figure 11. We can see that the change maps presented in column d are generated by baseline, and these change maps have many problems such as missing change regions and small target omission detection, which are extremely poor visual effects. Compared with baseline, the baseline+ A-R method has been able to initially detect the contours of some small road changes and fill in some of the missing change regions, which is due to our deep modification of the convolutional block of U-Net++, and further indicates that our proposed A-R significantly enhances the U-Net++ performance. The baseline+AFFM method effectively fuses the four levels of features output by backbone under the role of AFFM, so that the network has the ability to exploit both high- and low-level information and feature contextual association, and has been able to detect most of the changed regions, but there is still the problem of discontinuous changed regions. From column g we can see that the visual effect of the change map output by the baseline+HARM method has been basically the same as that of Ground Truth, but there is still the problem of unclear boundary when detecting some small changed objects. Therefore, baseline+AFFM+HARM and baseline+A-R +AFFM+HARM aim to solve the problems of discontinuous changed regions and unclear boundaries, etc. From the results in columns h and i, we can see that the change map is already very similar to the visual effect of Ground Truth, the detection of fine roads is more accurate and continuous, and the boundary delineation of changed regions is also more accurate. We also compare the attentional heat map generated by the baseline model UNet++ and our model HARNU-Net, as shown in Figure 12. It can be seen that our model focuses more accurately on the real change region than the baseline, effectively eliminating the effect of pseudo-change. By evaluating the visualization results of the above methods, the usefulness of each module proposed in this paper is effectively illustrated.

**Figure 13:**
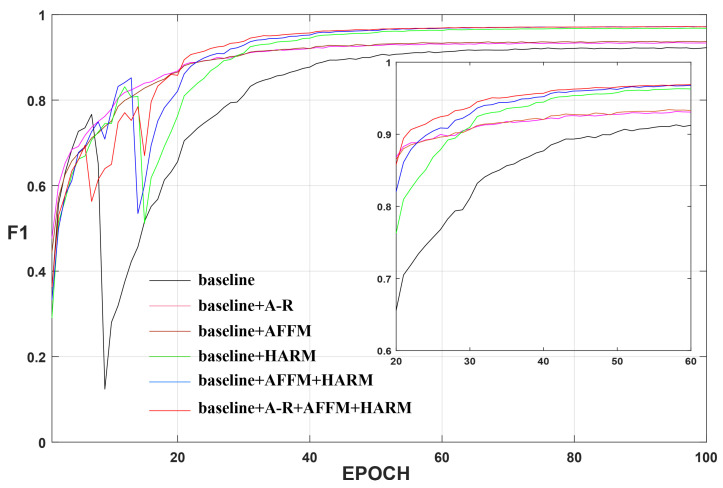
Validation results F1 of ablation experiments on CDD dataset. Smaller rectangular boxes show a clearer result.

In addition, we also counted and compared the evaluation metrics of these experiments in Table 5. The rightmost column indicates the speed of change map generation for the CDD test set when we applied different methods on a single NVIDIA Titan RTX. Comparing with baseline, the other five experiments have 0.45% to 4.29% and 0.78% to 7.79% improvement in the main metrics F1 and IoU, respectively. In Table 5, after improving or adding any of the modules, all metrics have corresponding improvements. After adding all modules to the baseline to form our HARNU-Net, F1 and IoU are improved by a maximum of 4.29% and 7.79%, respectively, which illustrates the innovation and effectiveness of our proposed three modules. Although our model achieves the best evaluation metrics, it is inferior in terms of visualization speed, the shortcoming that will be the focus of our subsequent research. We show a line graph of the validation results F1 for each ablation experiment, as shown in Figure 13. We can see that after about the 20th epoch, each experiment performs better than the baseline (black curve), and the validation results for our “complete body” (red curve) performs much better than the baseline.

### 4.5. Analysis of the Role of HARM

As our main innovation and a key component of HARNU-Net, we have made a detailed comparison and research on HARM to elaborate the rationality and usefulness of HARM in detail. Specifically, we compared HARM with several popular attention modules, studied the rationality of the hierarchical structure in HARM, and tested the effect of HARM acting on other networks. The specific experimental analysis is shown below.

#### 4.5.1. Attention Module Comparison

To investigate the importance of HARM for HARNU-Net, we compare HARM with several popular attention modules, namely CAM [44], SAM [44], CBAM [44], and SE [62]. We insert these attention modules into HARNU-Net to replace HARM, keeping the other structures and various hyperparameters intact, in order to compare which attention module contributes more to network. As observed from Table 6, the method applying HARM has 0.93% to 2.83% and 1.74% to 5.21% higher F1 and IoU, respectively, than the other methods. This confirms that our HARM is more reasonable than the direct application of various types of attention modules. With the help of HARM, our HARNU-Net can significantly overcome the interference of various types of pseudo-change and accurately capture the true position of the changed objects.

#### 4.5.2. Rationality of the Hierarchical Structure

**Figure 14:**
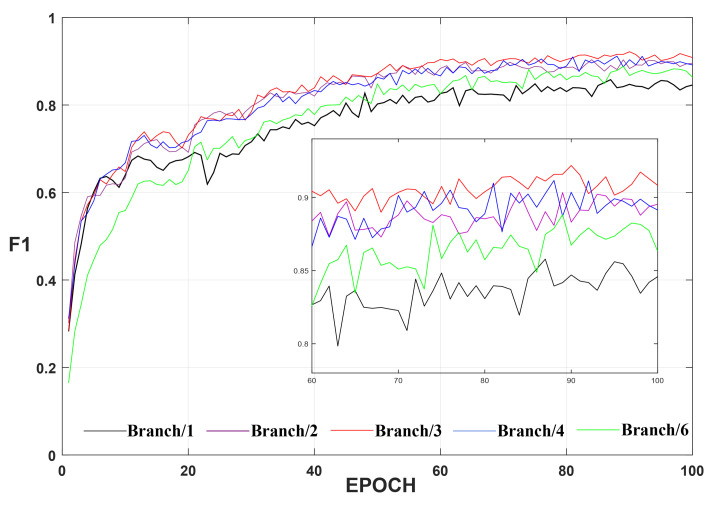
Validation results F1 for different branches of HARM on the BCDD dataset. Smaller rectangular boxes show a clearer result.

To validate the rationality of the hierarchical structure in HARM, we validated five branching schemes with 1, 2, 3, 4, and 6 branches on the BCDD dataset. Results presented in Table 7 indicate that the optimal performance is obtained when the number of branches is 3, after which the performance gradually decreases as the number of branches increases. However, multiple branches always outperforms single branches, which indicates that the transfer and interaction of information between hierarchies is contributing in enhancing the capability of HARM. The possible reason the performance starts to degrade after the number of branches reaches 3 is that the number of channels of features is fixed and limited, and each branch contains less channel information as the number of branches increases, which can cause the initial branches to be over-enhanced during the transfer of the hierarchy, resulting in the loss or distortion of useful information. Considering that a multi-branch module limited by the hierarchy would slow down the model, we only conducted experiments containing up to six branches. It was finally concluded that the best trade-off between the number of branches and the number of channels was achieved at a branch count of 3, resulting in the best performance. We also show a line graph of the validation results F1 for the model with different branches of HARM, as shown in Figure 14. The black curve represents the single-branch scheme, which always has lower validation results than the multi-branch scheme, where the red curve represents the best validation results for the three-branch scheme.

#### 4.5.3. Validity of HARM

**Figure 15:**
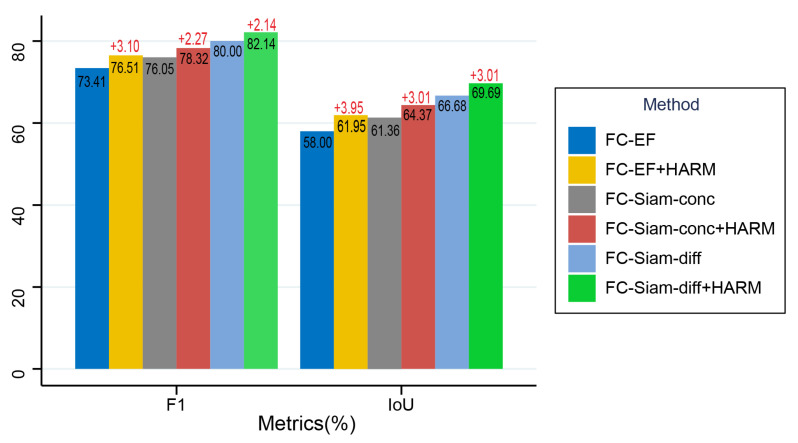
Ablation experiments performed on the LEVIR-CD dataset applying HARM to other models. All scores are expressed as a percentage (%).

To verify the effectiveness of HARM, we added HARM to three classical CD networks derived from FCN variants, FC-EF, FC-Siam-conc and FC-Siam-diff, and did experiments on the LEVIR-CD dataset for comparison with the original networks. From Figure 15 we can see that FC-EF increases F1 by 3.10% and IoU by 3.95% after adding HARM; FC-Siam-conc increases F1 by 2.27% and IoU by 3.01% after adding HARM; FC-Siam-diff increases F1 by 2.14% and IoU increased by 3.01% after adding HARM. This confirms that HARM can significantly enhance the performance of the CD network and help the network capture more changed objects. We further recognize the contribution of the hierarchical structure to HARM.

## 5. Conclusions

In this paper, we proposed a Hierarchical Attention Residual Nested U-Net (HARNU-Net) for remote sensing images CD. To enhance the capacity of the backbone network U-Net++, we proposed A-R by remodeling its convolutional blocks, and A-R helped the backbone network replenish the missing part of information in feature extraction. Meanwhile, in order to enhance the fusion of features at all levels, we used AFFM to fuse the features from the backbone network according to the adjacent strategy, which effectively realized the mutual enhancement between feature contexts. In addition, to strengthen the feature characterization at finer granularity, we proposed HARM to achieve the elimination of invalid information of features at the channel and spatial dimensions at a finer granularity space, which provided the output of change maps with better visual effects. In our experiments, we compared HARNU-Net with seven other state-of-the-art CD methods on three CD datasets, and our method yielded the best results in terms of metrics and visualization results. Finally, we validated the usefulness of each module by ablation experiments. However, in our study, we found that our model has a disadvantage in terms of speed, so in the subsequent work our research focus will be on the implementation of model lightweighting and high-speed.

## Figures and Tables

**Table 1:** Input and output size of the backbone network.

A-R	Size (Channel × Height × Width)	
	Input	Output
$X_{A}^{0,0}$	3 × 256 × 256	48 × 256 × 256
$X_{B}^{0,0}$	3 × 256 × 256	48 × 256 × 256
$X^{0,1}$	192 × 256 × 256	48 × 256 × 256
$X^{0,2}$	240 × 256 × 256	48 × 256 × 256
$X^{0,3}$	288 × 256 × 256	48 × 256 × 256
$X^{0,4}$	336 × 256 × 256	48 × 256 × 256
$X_{A}^{1,0}$	48 × 128 × 128	96 × 128 × 128
$X_{B}^{1,0}$	48 × 128 × 128	96 × 128 × 128
$X^{1,1}$	384 × 128 × 128	96 × 128 × 128
$X^{1,2}$	480 × 128 × 128	96 × 128 × 128
$X^{1,3}$	576 × 128 × 128	96 × 128 × 128
$X_{A}^{2,0}$	96 × 64 × 64	192 × 64 × 64
$X_{B}^{2,0}$	96 × 64 × 64	192 × 64 × 64
$X^{2,1}$	768 × 64 × 64	192 × 64 × 64
$X^{2,2}$	960 × 64 × 64	192 × 64 × 64
$X_{A}^{3,0}$	192 × 32 × 32	384 × 32 × 32
$X_{B}^{3,0}$	192 × 32 × 32	384 × 32 × 32
$X^{3,1}$	1536 × 32 × 32	384 × 32 × 32
$X_{B}^{4,0}$	384 × 16 × 16	768 × 16 × 16

$X_{A(B)}^{i,j}$: i, j denote the i-th and j-th levels of the encoder and decoder, respectively, and A(B) denotes the processing of the A(B)-temporal picture in Figure 1a.

**Table 2:** Main indicators of the three dataset we used.

Datasets	Resolution	Size	Number of Pixels			Change Objects	Number of Samples		
			Changed	Unchanged	Ratio		Train	Validation	Test
CDD	0.03–1 m/pixel	256 × 256	134,068,750	914,376,178	1:6.82	buildings, roads, cars, etc.	10,000	3000	3000
BCDD	0.3 m/pixel	256 × 256	21,352,815	477,759,663	1:22.37	Sparse large buildings	5948	743	743
LEVIR-CD	0.5 m/pixel	256 × 256	30,913,975	637,028,937	1:20.61	Dense small buildings	7120	1024	2048

**Table 3:** Key characteristics of comparative methods.

Models	Year	Architecture	Main Strategy	Loss Function
FC-EF	2018	Single-stream, FCN	Skip connection, multi-level fusion	WSCE loss ^1^
FC-Siam-conc	2018	Siamese, FCN	Siamese-concatenation, skip connection, multi-level fusion	WSCE loss
FC-Siam-diff	2018	Siamese, FCN	Siamese-difference, skip connection, multi-level fusion	WSCE loss
CDNet	2018	Single-stream, FCN	Stacking contraction, expansion blocks	Weighted cross-entropy loss
STANet	2020	Siamese, ResNet	BAM,PAM	Batch-balanced contrastive loss
BIT	2021	Siamese, ResNet	Bi-temporal image transformer	Cross-entropy loss
SNUNet	2021	Siamese, UNet++	Densely connected, ECAM	WSCE loss, dice loss

^1^ represent Weighted Softmax Cross-Entropy loss.

**Table 4:** Quantitative results on the three dataset. The best two results are in bold and underline. All scores are expressed as a percentage (%).

Model	CDD	BCDD	LEVIR-CD
	Pre/Recall/F1/IoU/OA	Pre/Recall/F1/IoU/OA	Pre/Recall/F1/IoU/OA
FC-EF	76.56/43.49/55.47/38.38/91.76	82.28/70.66/76.03/61.33/97.92	82.27/66.28/73.41/58.00/97.55
FC-Siam-conc	88.00/53.58/66.61/49.93/93.66	40.09/73.84/51.97/35.11/93.63	86.81/67.66/76.05/61.36/97.83
FC-Siam-diff	88.49/51.53/65.14/48.30/93.49	38.82/71.80/50.40/33.69/93.40	86.55/74.38/80.00/66.68/98.11
CDNet	91.93/84.87/88.26/78.98/97.34	92.16 /83.18/87.44/77.68/98.88	88.38/85.08/86.70/76.52/98.67
STANet	88.97/94.31/91.56/84.44/97.95	91.25/86.18/88.64/79.61/98.97	80.99/**91.21**/85.79/75.12/98.46
BIT	95.86/94.59/95.22/90.88/98.88	86.07/85.61/85.84/75.19/98.68	**91.95** /88.57/90.23/82.19/**99.02**
SNUNet	96.82/96.72/96.77/93.74/99.24	88.35/87.80/88.07/78.69/98.89	91.66/88.48/90.04/81.89/99.00
Ours	**97.10**/**97.30**/**97.20**/**94.56**/**99.34**	**92.70**/**88.72**/**90.67**/**82.93**/**99.15**	91.23/89.37/**90.29**/**82.30**/**99.02**

**Table 5:** Ablation experiments of the modules in our model on the CDD dataset and the speed of change map generation. The highest score is marked in bold. The five evaluation metrics are expressed in percentage (%).

Baseline	A-R	AFFM	HARM	CDD					
				Pre	Recall	F1	IoU	OA	Sheets/Sec.
✓				94.99	90.93	92.91	86.77	98.36	**41.10**
✓	✓			94.77	92.00	93.36	87.55	98.46	21.28
✓		✓		95.32	93.31	94.31	89.22	98.67	38.96
✓			✓	96.70	96.66	96.68	93.57	99.22	29.70
✓		✓	✓	96.94	97.06	97.00	94.17	99.29	26.41
✓	✓	✓	✓	**97.10**	**97.30**	**97.20**	**94.56**	**99.34**	18.40

✓ indicates that the method is used in this experiment.

**Table 6:** Ablation experiments performed on the CDD dataset applying different attention modules to our model. The highest score is marked in bold. All scores are expressed as a percentage (%).

	CDD				
	Pre	Recall	F1	IoU	OA
**CAM**	95.17	93.58	94.37	89.35	98.68
**SAM**	95.5	94.01	94.75	90.02	98.77
**CBAM**	96.18	94.6	95.38	91.17	98.92
**SE**	96.71	95.84	96.27	92.82	99.13
**HARM**	**97.1**	**97.3**	**97.2**	**94.56**	**99.34**

**Table 7:** Ablation Experiment of rationality analysis of the hierarchical structure in HARM on the BCDD dataset. The highest score is marked in bold. All scores are expressed as a percentage (%).

HARM_ Branch	BCDD				
	Pre	Recall	F1	IoU	OA
**1**	88.87	84.94	86.86	76.77	98.8
**2**	90.64	86.08	88.3	79.05	98.94
**3**	**92.7**	88.72	**90.67**	**82.93**	**99.15**
**4**	86.22	**88.77**	87.47	77.75	98.81
**6**	89.92	86.1	87.97	78.53	98.9

## Data Availability

The CDD, BCDD, LEVIR-CD datasets are openly available at https://drive.google.com/fifile/d/1GX656JqqOyBi_Ef0w65kDGVto-nHrNs9 (accessed on 16 May 2022), http://gpcv.whu.edu.cn/data/building_dataset.html (accessed on 16 May 2022), https://justchenhao.github.io/LEVIR/ (accessed on 16 May 2022), respectively.
